# Preservation of Distortion Product Otoacoustic Emission After Cochlear Implantation in Children With *OTOF*‐Related Deafness

**DOI:** 10.1002/mco2.71006

**Published:** 2026-09-09

**Authors:** Chang‐Hee Kim, Bong Jik Kim, Ja‐Won Koo, Ngoc‐Trinh Tran, Yehree Kim, Byung Yoon Choi

**Affiliations:** ^1^ Department of Otorhinolaryngology—Head and Neck Surgery Konkuk University Medical Center Research Institute of Medical Science Konkuk University School of Medicine Seoul Republic of Korea; ^2^ Climate‐Environment‐Society‐Digital Innovation (CSI) Lab Chungnam National University Daejeon Republic of Korea; ^3^ Department of Otorhinolaryngology‐Head and Neck Surgery Seoul National University College of Medicine and Seoul National University Bundang Hospital Seongnam‐si South Korea

1

Dear Editor,

The emergence of adeno‐associated virus (AAV)–mediated gene therapy for *OTOF*‐related deafness (DFNB9) marks a historic milestone in otology, offering the first biological cure for congenital hearing loss. Current clinical trials (e.g., NCT05788536, NCT05821959) have shown promising efficacy in restoring auditory thresholds, with some participants achieving near‐normal hearing levels [1, 2, 3]. However, the long‐term effects of gene therapy on speech and language development remain incompletely understood [1]. In contrast, the benefits of early cochlear implantation (CI) are well established. Early CI, combined with appropriate rehabilitation, promotes normal cortical auditory development and supports age‐appropriate language acquisition in young children.

Given the importance of timely auditory access during early development, the postoperative cochlear physiologic status of children with *OTOF*‐related deafness after CI may be clinically relevant to characterize. Although CI currently provides a well‐established intervention with extensive long‐term outcome data, it requires implantation of a permanent device and may be associated with surgical risks, device dependence, or MRI‐related imaging limitations. In contrast, gene therapy offers the potential for biological restoration without implanted hardware, and questions regarding long‐term durability, patient eligibility, and accessibility are currently under active investigation. Current gene therapy trials predominantly recruit CI‐naïve infants, leaving uncertainty about whether children who have already received CI might still be candidates for future biological interventions. This question is particularly relevant given DFNB9 pathophysiology: unlike typical hair cell degeneration, *OTOF* variants cause synaptic dysfunction while often sparing outer hair cells (OHCs) and cochlear gross morphology.

This study aims to describe postoperative distortion product otoacoustic emission (DPOAE) findings, and investigate if OHC function is preserved in children with *OTOF*‐related deafness after CI.

In this cross‐sectional analysis of a retrospective cohort, we evaluated 11 children with confirmed biallelic *OTOF* variants who underwent CI at Seoul National University Bundang Hospital. Postoperative DPOAEs were assessed across a range of CI durations using a cross‐sectional “space‐for‐time substitution” approach. Detailed information on the cohort, DPOAE assessment, and statistical analysis is provided in the Supporting Information.

Detectable OHC function appeared to be associated with both age at implantation and duration of CI use. Ears with detectable DPOAEs were generally those implanted at younger ages and assessed at shorter postoperative intervals. Specifically, among 5 infants implanted at ≤ 12 months and evaluated within 6 years, detectable OHC function was observed in 7 of 10 ears (Figure 1B,C). None of the 12 ears from the remaining comparison group (implanted > 12 months or duration ≥ 6 years) showed preservation. This difference was significant on Fisher's exact test (*p* = 0.001). When accounting for bilateral ears from the same patient using generalized estimating equations (GEE) with an exchangeable correlation structure, the association remained significant (GEE, *p* < 0.001). However, the small sample size and limited age range preclude any definitive conclusion about a specific age threshold for OHC preservation. Overall, detectable postoperative OHC function was observed predominantly in ears implanted at a younger age and evaluated within a shorter duration after CI.

**FIGURE 1:**
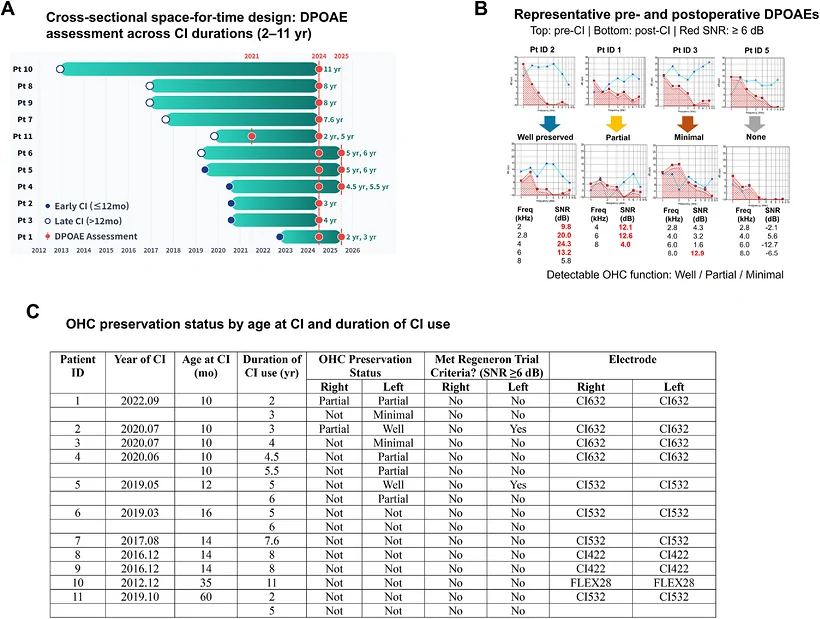
Evaluation of post‐cochlear implantation (CI) distortion product otoacoustic emission (DPOAE) preservation via space‐for‐time substitution. (A) The “space‐for‐time” substitution design. To overcome the prohibitive latency of prospective longitudinal studies, this study employed a cross‐sectional “space‐for‐time” substitution strategy. By assessing a cohort with heterogeneous CI durations (2–11 years) at a single time point (2024–2025), we effectively reconstructed the longitudinal trajectory of outer hair cell (OHC) survival. This model operates on the premise that ears with longer CI durations represent the “future state” of recently implanted ears, enabling the delineation of the long‐term course of residual OHC function, as assessed by DPOAEs, without the need for decades of longitudinal observation. (B) Evidence of cochlear structural integrity post‐CI longitudinal comparison of DPOAEs in four representative *OTOF*‐related hearing loss (DFNB9) CI cases. Top row: preoperative baseline responses. Middle row: postoperative responses obtained from the implanted ear, demonstrating a spectrum of preservation outcomes from “well preserved” to “none.” Bottom row: quantitative signal‐to‐noise ratio (SNR) data for the postoperative state. Values in red indicate an SNR ≥ 6 dB, the threshold for robust OHC function. This confirms that modern atraumatic CI surgery may preserve OHC function (e.g., Pt ID 2 and 1). (C) OAE‐based OHC preservation status after CI. This panel summarizes 11 DFNB9 children with prior CI, including age at CI, CI duration, implanted electrode type, OHC preservation status (right/left), and whether each ear met the Regeneron DPOAE criterion (SNR ≥ 6 dB).

Taken together, detectable postoperative cochlear physiologic signals were observed in a subset of children with *OTOF*‐related deafness after CI in our cohort. Progressive loss of residual hearing has been reported after hearing‐preservation CI with hearing preservation electrode [4], and accordingly, the present study cannot determine whether reduced postoperative DPOAEs in a subset of implanted ears reflect natural disease‐related progression, implantation‐related effects, or both. Meanwhile, our findings are consistent with Santarelli et al. [5], who showed that DPOAEs in *OTOF*‐related hearing loss may persist much longer than previously thought and that middle ear effusion can lead to underestimation of OAE preservation. In their cohort, DPOAEs remained detectable during long‐term follow‐up in several patients, including in the implanted ear of Patient 8 at 6.4 years of age, approximately 3.6 years after implantation at 33 months. Importantly, their main message was not the low overall rate of OAE preservation after implantation, but the persistence of detectable OHC function for many years, even in implanted ears. They also suggested that some reported OAE loss may have reflected transient middle ear pathology rather than true OHC deterioration. Although most implanted subjects in their study received thicker electrodes (CI24RE(CA) or CI512) and were followed longer than in the present study, DPOAEs were still preserved in one patient. Therefore, the persistence of DPOAEs in our cohort, mostly implanted with contemporary slim electrodes and evaluated within 6 years after implantation, is not discrepant with their findings. Rather, both studies suggest that measurable OHC function can persist for years in a subset of implanted *OTOF* ears after CI.

Our findings indicate that DPOAEs remained detectable in a subset of implanted ears. However, because DPOAEs primarily assess OHC‐mediated cochlear amplifier function, the presence or absence of DPOAEs cannot be assumed to signify overall cochlear integrity. For instance, patients with *STRC*‐related hearing loss may have absent DPOAEs yet retain preserved speech discrimination, illustrating that the absence of DPOAEs does not necessarily reflect non‐functional inner hair cell or organ of Corti function. Further studies are needed to determine the clinical relevance of preserved DPOAEs following CI. Although the implications of present findings remain uncertain, they may serve as a reference for future investigations as gene therapy and other biological treatments for hearing loss continue to evolve.

## Author Contributions

Conceptualization: Byung Yoon Choi, Chang‐Hee Kim, and Bong Jik Kim; Methodology: Byung Yoon Choi, Chang‐Hee Kim, and Bong Jik Kim; Investigation: Chang‐Hee Kim, Bong Jik Kim, Ja‐Won Koo, Ngoc‐Trinh Tran, Yehree Kim, and Byung Yoon Choi; Formal analysis: Chang‐Hee Kim, Bong Jik Kim, Ja‐Won Koo, Ngoc‐Trinh Tran, Yehree Kim, and Byung Yoon Choi; Data curation: Chang‐Hee Kim, Bong Jik Kim, Ja‐Won Koo, Ngoc‐Trinh Tran, Yehree Kim, and Byung Yoon Choi; Writing – original draft: Byung Yoon Choi, Chang‐Hee Kim, and Bong Jik Kim; Writing – review and editing: Byung Yoon Choi; Supervision: Byung Yoon Choi. Byung Yoon Choi had full access to all the data in the study and takes responsibility for the integrity of the data and the accuracy of the data analysis. All authors have read and approved the final manuscript.

## Ethics Statement

This study was conducted in accordance with the Declaration of Helsinki and was approved by the Institutional Review Board of Seoul National University Bundang Hospital (Approval No. B‐2601‐1023‐111). The requirement for informed consent was waived by the Institutional Review Board because of the retrospective nature of the study.

## Conflicts of Interest

The authors declare no conflicts of interest.

## Supporting information




**Supporting File: 1** mco271006‐sup‐0001‐SuppMat.docx

## Data Availability

The data may be available from the corresponding author upon reasonable request and with approval from the relevant institutional review board.
