# Supplementary material for: Preservation of Distortion Product Otoacoustic Emission After Cochlear Implantation in Children With OTOF‐Related Deafness
Source: MedComm (2020). 2026 Sep 9;7(9):e71006. doi: 10.1002/mco2.71006 (PMC13555795; doi:10.1002/mco2.71006)
Supplement: Supplementary file 1 [file MCO2-7-e71006-s001.docx]

**Preservation of distortion product otoacoustic emission after cochlear implantation in children with *OTOF*-related deafness**

Chang-Hee Kim, MD, PhD^1#^, Bong Jik Kim, MD, PhD^2#^,  [Ja-Won Koo](https://pubmed.ncbi.nlm.nih.gov/?sort=date&term=Koo+JW&cauthor_id=34373484), MD, PhD^3^, [Ngoc-Trinh Tran](https://pubmed.ncbi.nlm.nih.gov/?sort=date&term=Tran+NT&cauthor_id=40515814), PhD^3^, Yehree Kim, MD, PhD^3^, Byung Yoon Choi, MD, PhD^3*^

^1^Department of Otorhinolaryngology-Head and Neck Surgery, Konkuk University Medical Center, Research Institute of Medical Science, Konkuk University School of Medicine, Seoul, Republic of Korea

^2^Climate-Environment-Society-Digital Innovation (CSI) Lab, Chungnam National University, Daejeon, Republic of Korea

^3^Department of Otorhinolaryngology-Head and Neck Surgery, Seoul National University College of Medicine and Seoul National University Bundang Hospital, 82 Gumi-ro 173 beon-gil, Bundang-gu, Seongnam-si, Gyeonggi-do 13620, South Korea

^#^These two authors contributed equally to this project.

**Running title**: DPOAE Preservation after CI in *OTOF* Deafness

**^*^Correspondence to** Byung Yoon Choi, M.D., Ph.D.

Department of Otorhinolaryngology, Seoul National University Bundang Hospital, Seoul National University College of Medicine, 82, Gumi-ro 173beon-gil, Bundang-gu, Seongnam-si, Gyeonggi-do 13620, Republic of Korea.

**E-mail:** choiby2010@gmail.com; [twinwif2@snu.ac.kr](mailto:twinwif2@snu.ac.kr); choiby@snubh.org, **Tel:** +82-31-787-7406, **Fax:** +82-31-787-4057

**Study design and cohort**

This study was a cross-sectional analysis of a retrospective hospital-based cohort of children with genetically confirmed *OTOF*-related hearing loss (DFNB9) treated at Seoul National University Bundang Hospital (SNUBH), a tertiary referral hospital in Seongnam, Gyeonggi-do, Republic of Korea. We identified 11 children with confirmed biallelic *OTOF* variants who underwent cochlear implantation (CI) with either slim straight or slim modiolar electrode arrays between 2012 and 2022 at SNUBH. The duration of CI use at postoperative distortion product otoacoustic emission (DPOAE) assessment ranged from 2 to 11 years. The cohort was derived from patients treated at SNUBH; no community-based cohort was included.

Clinical and surgical records were retrospectively reviewed. Surgical records confirmed that all implantations were performed using soft-surgery techniques designed to preserve residual hearing and minimize intracochlear trauma.

**Cross-sectional postoperative assessment**

Routine serial postoperative DPOAE monitoring was not part of the standard clinical protocol. Therefore, we used a cross-sectional "space-for-time substitution" approach and performed a comprehensive single-time-point audiological assessment of the participants during the study period. By including participants with heterogeneous durations of CI use, this approach allowed descriptive comparison of postoperative DPOAE status across different postoperative intervals. Because the observations were cross-sectional, this design was not intended to establish individual longitudinal trajectories of outer hair cell (OHC) function.

**DPOAE assessment and classification**

DPOAEs were measured at f2 frequencies from 1 to 8 kHz. Preservation criteria were aligned with the Regeneron DB-OTO trial (NCT05788536) criteria: (1) well preserved, signal to noise ratio (SNR) ≥6 dB at ≥3 frequencies; (2) partially preserved, SNR ≥6 dB at 2 frequencies or ≥5 dB at ≥3 frequencies; (3) minimally preserved, SNR ≥6 dB at 1 frequency; and (4) not preserved. For the present analysis, "detectable OHC function" was defined as meeting the criteria for well preserved, partially preserved, or minimally preserved DPOAEs.

**Statistical analysis**

The analysis was performed at the ear level. Categorical data were summarized as counts and percentages. Given the small sample size, Fisher's exact test was used to compare the proportion of ears with detectable OHC function between ears from children implanted at ≤12 months of age and assessed within 6 years after CI and the remaining ears (implantation at >12 months of age or CI duration ≥6 years). Because two ears from the same child could contribute observations, generalized estimating equations (GEE) with an exchangeable working correlation structure were additionally used to account for within-participant correlation between bilateral ears. Statistical tests were two-sided, and *P* < 0.05 was considered statistically significant.

The age-at-implantation and CI-duration grouping was used for exploratory comparison in this small cohort; the analysis was not designed to establish a definitive age or postoperative-duration threshold for DPOAE preservation.

**Ethics**

This study was conducted in accordance with the Declaration of Helsinki and was approved by the Institutional Review Board of Seoul National University Bundang Hospital (Approval No. B-2601-1023-111). The requirement for informed consent was waived by the Institutional Review Board because of the retrospective nature of the study.
